# Selection and Validation of Reference Genes for qRT-PCR in *Cycas elongata*

**DOI:** 10.1371/journal.pone.0154384

**Published:** 2016-04-28

**Authors:** Yanting Hu, Tian Deng, Letian Chen, Hong Wu, Shouzhou Zhang

**Affiliations:** 1 State Key Laboratory for Conservation and Utilization of Subtropical Agro-Bioresources, South China Agricultural University, Guangzhou, Guangdong, China; 2 Fairylake Botanical Garden, Shenzhen and Chinese Academy of Sciences, Shenzhen, Guangdong, China; Wuhan Botanical Garden, Chinese Academy of Sciences, Wuhan, China, CHINA

## Abstract

Quantitative reverse transcription PCR (qRT-PCR) is a sensitive technique used in gene expression studies. To achieve a reliable quantification of transcripts, appropriate reference genes are required for comparison of transcripts in different samples. However, few reference genes are available for non-model taxa, and to date, reliable reference genes in *Cycas elongata* have not been well characterized. In this study, 13 reference genes (*ACT7*, *TUB*, *UBQ*, *EIF4*, *EF1*, *CLATHRIN1*, *PP2A*, *RPB2*, *GAPC2*, *TIP41*, *MAPK*, *SAMDC* and *CYP*) were chosen from the transcriptome database of *C*. *elongata*, and these genes were evaluated in 8 different organ samples. Three software programs, NormFinder, GeNorm and BestKeeper, were used to validate the stability of the potential reference genes. Results obtained from these three programs suggested that *CeGAPC2* and *CeRPB2* are the most stable reference genes, while *CeACT7* is the least stable one among the 13 tested genes. Further confirmation of the identified reference genes was established by the relative expression of *AGAMOUSE* gene of *C*. *elongata* (*CeAG*). While our stable reference genes generated consistent expression patterns in eight tissues, we note that our results indicate that an inappropriate reference gene might cause erroneous results. Our systematic analysis for stable reference genes of *C*. *elongata* facilitates further gene expression studies and functional analyses of this species.

## Introduction

Quantitative reverse-transcription PCR (qRT-PCR) has been a key technology of gene expression analysis in numerous molecular biology applications [1]. With high throughput capability for quantification of transcript levels, qRT-PCR is considered highly sensitive, accurate, and reproducible [2]. However, some variables such as the integrity, amount and purity of the RNA, as well as enzyme efficiency during cDNA synthesis and PCR amplification make it necessary to normalize the data for an accurate and reliable result [3]. Thus, a reliable reference gene in which expression is constant and stable at different developmental stages, nutritional conditions or experimental conditions is required for normalization [4]. Common reference genes or internal control genes used in qRT-PCR are housekeeping genes (HKGs) related to cell maintenance, such as *actin* (*ACT*), *tubulin* (*TUB*), *glyceraldehyde-3-phosphate dehydrogenase* (*GAPDH*), *18S ribosomal RNA* (*18S*), *ubiquitin* (*UBQ*) and *elongation factor 1-α* (*EF 1-α*) [5–7]. New reference genes have been studied in model and non-model plants, such as *Arabidopsis* [7], African oil palm [8] and Brazilian pine [5]. However, a comprehensive genome sequence of *Cycas elongata* is not yet available since identification of internal control genes is time consuming and specialized work. Therefore, the selection of reference genes for qRT-PCR has not yet been reported in *C*. *elongata*.

In the last two decades, progress has been made in our understanding of the molecular mechanisms of floral developmental control in angiosperms [9]. Development of eudicot flowers is controlled by MADS box genes in a well-known ABCDE model [10]. However, it is still largely unclear how the flower originated during evolution [11–13]. In a comprehensive framework of evolutionary developmental biology (‘evo-devo’), the origin and evolution of the gene controlling floral organ specification is intimately intermingled with the evolutionary origin and diversification of the angiosperm flower [14]. To better understand the origin of the flower, it is inevitable to study gymnosperms, the closest extant relatives of the angiosperms. Extant gymnosperms comprise conifers, gnetophytes, Ginkgo and cycads. Only one survey of cycads regarding the MADS-box gene [15] has been described, wheras several studies about MADS-box genes of conifers, gnetophytes and Ginkgo have been reported [13, 14, 16]. Thus, it is necessary to select stable reference genes of cycads for further molecular studies.

In this current study, thirteen candidate reference genes were evaluated in eight plant tissues (megasporophyll, microsporophyll, ovules, roots, stalks, female plant leaves, male plant leaves and asexual plant leaves). Based on the transcriptome data of *C*. *elongata* (unpublished), the thirteen candidate reference genes were first identified by their orthologous genes in model plants, then cloned, sequenced and confirmed. To validate our results, we used the most stable reference genes to assess the expression levels of *CeAG* gene in the eight plant tissues.

## Materials and Methods

### Plant materials

**Fig 1:**
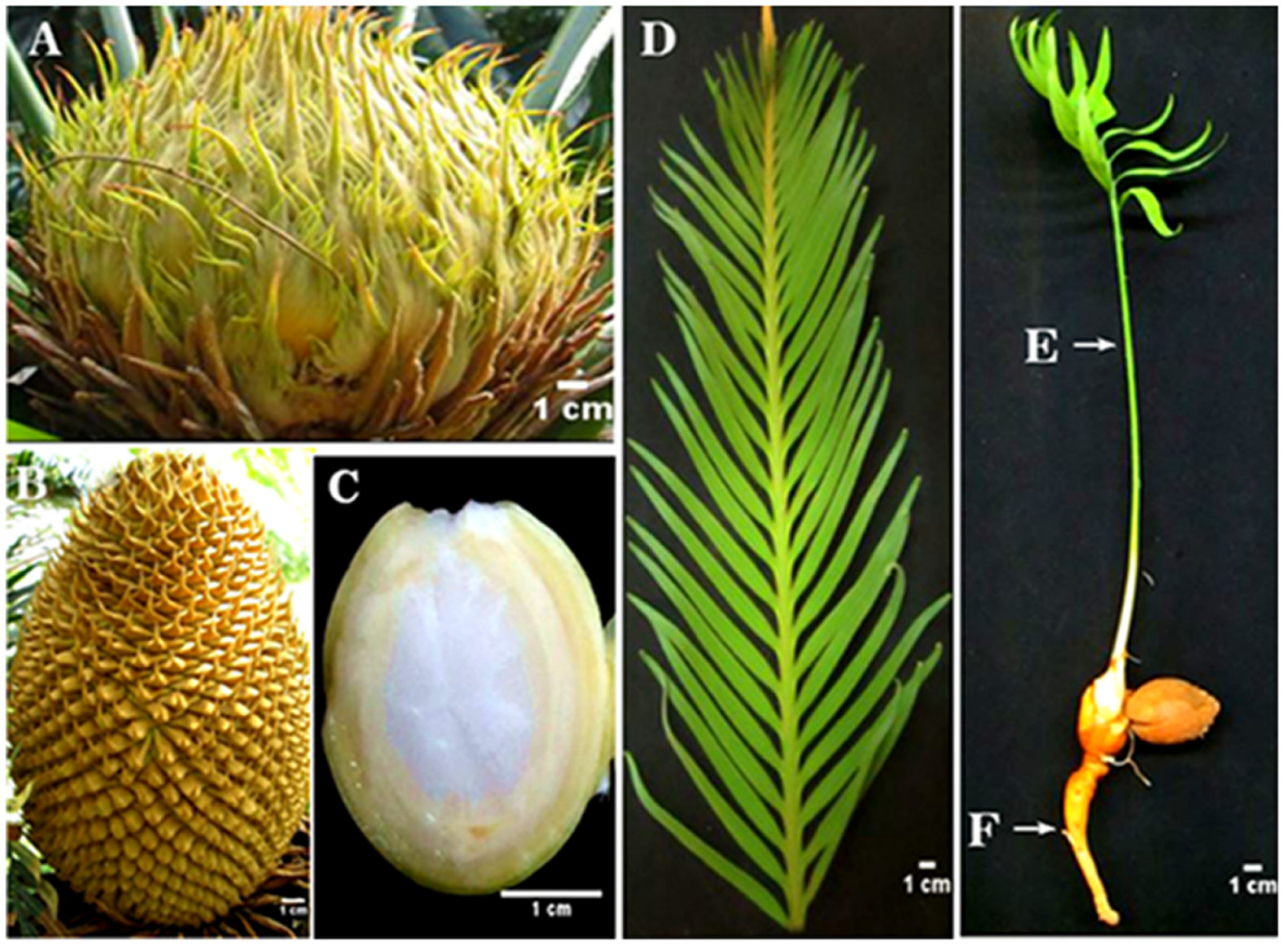
*C*. *elongata* tissues and organ sample set. (A) Megasporophyll; (B) Microsporophyll; (C) Ovule; (D) Leaf; (E) Stalk; (F) Root. Scale bars = 1 cm.

Reproductive and vegetative tissues were obtained from the female, male and asexual *C*. *elongata* plants growing in Cycads Conservation Center in the Fairylake Botanical Garden. The megasporophyll, microsporophyll, ovule and leaves of female plants, male plants and asexual plants were harvested from *C*. *elongate* specimens that were approximately twenty years old (Fig 1A, 1B, 1C and 1D). The root and stalk were collected from seedlings (Fig 1E and 1F) and washed through with deionized water, and then subjected to a one minute sterilization with 75% alcohol. Each type of harvested tissues were divided in two biological replicates and all of them were flash-frozen in liquid nitrogen and stored at -80°C until needed.

### Total RNA extraction and cDNA synthesis

One gram of frozen tissue samples were grounded to fine powder with mortar and pestle in liquid nitrogen. To extract total RNA we used a Magen HiPure Plant RNA Kit (http://www.magentec.com.cn/products.php?ID=504) per the manufacturer’s instructions. We determined concentration, purity, and integrity of the RNA samples using an ultraviolet spectrophotometer (TU-1810, PGENERAL, China) and visualized *via* gel electrophoresis (2% agarose) (S1 Fig). The samples with 260/280 nm and 260/230 nm ratios between 1.9–2.5 and 1.9–2.2 (S2 Table), were considered for use in subsequent analyses. We used 2 μg of RNA for each sample used in genomic DNA elimination and reverse-transcription in order to acquire cDNA for RT-PCR. These processes were accomplished with the PrimeScript^™^ RT reagent Kit with gDNA Eraser (TaKaRa, Inc). The cDNA solution was diluted 20 times with EASY Dilution (TaKaRa, Inc) and aliquots were stored at -20°C until qRT-PCR.

### Candidate gene selection, PCR primer design and candidate gene cloning and multiple sequences alignments

We selected thirteen candidate reference genes based on previous studies [17, 18]. The BLAST program (E < 1e-10) was employed to survey the *C*. *elongata* transcriptome databases using the corresponding *Arabidopsis thaliana* protein sequences as query sequences (Table 1). We designed primers using the Primer Premier 5 program (http://www.premierbiosoft.com/index.html), and designed the primers to flank the conserved domains determined by an NCBI conserved domain search (http://www.ncbi.nlm.nih.gov/Structure/cdd/wrpsb.cgi). The target amplified fragments were excised from electrophoresis gel and purified with HiPure Gel Pure DNA Kits (Magen, China) and subcloned into the *pEASY*^®^-T1 Simple Cloning vector (the *pEASY*^®^-T1 Simple Cloning Vector Kit from TRANSGEN BIOTECH, China) following the guidance provided by the manufacturer. Plasmids were transferred into *Trans1*-T1 Phage Resistant Chemically competent cells (TRANSGEN BIOTECH, China) and recombinant colonies were sequenced by Life Technologies Company. The nucleotide sequences were analyzed by BLAST [19] against NCBI non redundant sequences (http://blast.ncbi.nlm.nih.gov).

**Table 1:** Candidate reference genes, annotated functions, and primers used to amplify and their PCR parameters.

Gene	*A*. *thaliana* Gene Description	*A*. *Thaliana* Ortholog	Primer sequences (5’ - 3’) forward/reverse	Tm (°C)	Amplicon length (bp)	Identity (%)
*CeCLATHRIN1*	clathrin adaptor complexes medium subunit family protein	NP_199475.1	CTTCACCACTACTCCCAATG/CCTGATGTCTTCAAAAGGGA	54	415	90
*CePP2A*	serine/threonine-protein phosphatase PP2A-1 catalytic subunit	NP_176192.1	GTTATCAAGCGTGTCCAAAG/GAATGTGCAACCTGTGAAAT	54	409	82
*CeRPB2*	DNA-directed RNA polymerase II subunit RPB2	NP_193902.1	TCTTTTGCAGGTAAGACCTC/ACGAAGATTATGTGGACGAG	52	1248	80
*CeGAPC2*	glyceraldehyde3-phosphate dehydrogenase GAPC2	NP_001077530.1	GGCCATTCCAGTCAATTTTC/GTCATCCATGAAAGGTTTGG	54	210	94
*CeTIP41*	TIP41-like protein tonoplast intrinsic protein4(Zea mays)	NP_195153.2	AGACAGCTCTCTTGAACTTG/TGAAATGCAAACCAGAATGG	55	900	84
*CeMAPK*	mitogen-activated protein kinase 1	NP_172492.1	GTGATTCTCTTACAGGGGTC/TCATACGGTATCGTCTGTTC	54	803	77
*CeSAMDC*	S-adenosylmethionine decarboxylase	NP_001154585.1	AAACCTTAGAAGCCCACAAT/TGTGTTAAGTACACTCGTGG	54	735	74
*CeEIF4*	eukaryotic initiation factor 4A-3	NP_177417.1	AACCTTCCGAGCTGTATTTT/TCCAGTCCTCCTTATCAACA	52	849	84
*CeEF1*	elongation factor 1-alpha 2	NP_563800.1	CAGCGAATTTTGAGAAGTGG/GGAACTCATACGAAGTCAGA	55	745	78
*CeACT7*	actin 7	NP_196543.1	GGAGGTGCTACAACCTTTAT/TGATGGAGTTACACACACTG	53	531	99
*CeTUB*	tubulin alpha-6 chain	NP_193232.1	GTTGGCCTCTCAATATCCAA/AATCCCTCTTCAGACCTCAT	55	731	80
*CeUBQ*	ubiquitin 11	NP_567286.1	GACGGGAAAGACCATAACTT/TGTGAATATAAGCCAGCGAT	52	400	81
*CeCYP*	Cyclophilin peptidyl-prolyl cis-trans isomerase CYP19-2	NP_179709.1	AAAATCCCAGGGTGTTCTTG/ATTTCACCGCTGTCTACAAT	55	511	81

### qRT-PCR primer design, quantitative real-time PCR and data acquisition

The primers for qRT-PCR were based on the the thirteen cloned *C*. *elongata* sequences and were designed using the Primer designing tool program (http://www.ncbi.nlm.nih.gov/tools/primer-blast/index.cgi?LINK_LOC=BlastHome). The primers exhibited melting temperatures between 57 and 59°C, primer lengths of 18–24 bp, GC content of 45–55% and amplicon lengths between104 and 287 bp. All qRT-PCR reactions were performed using a CTX96 Touch^™^ System (BIO-RAD) machine under following conditions: 3 min at 95°C, 36 cycles of 10 s at 95°C, 30 s at 58°C and 10 s at 72°C in 96 well plates. The qRT-PCR procedure concluded with a melt-curve ramping from 60 to 95°C for 20 min. The 20 μl reaction mixtures contained 4 μl of 20 fold diluted template, 2 μl of each amplification primer (10 μM), 10μl of iQ^™^ SYBR^®^ Green Supermix (BIO-RAD) and 4 μl sterile nuclease free water. All qRT-PCR reactions were carried out in technical triplicate and a non-template control was also included for each primer pair. Confirmation of primer specificity was based on the dissociation curve. In addition to baseline and threshold cycles (Ct), correlation coefficients (R^2^ values) and amplification efficiencies (E) for each primer pair was automatically determined using the CFX Manager^™^ Software (BIO-RAD). We used five dilutions of cDNA samples from *C*. *Elongata* vegetative leaves to obtain the standard curve.

### Analysis of gene expression stability

Three different types of Microsoft Excel-based software, geNorm [20], NormFinder [21] and BestKeeper [22], were used to evaluate the stability of reference gene expression. BestKeeper utilizes the method mean Ct values as input whereas geNorm and NormFinder convert Ct values using the formula: 2^−ΔCt^, where ΔCt = each corresponding Ct value—minimum Ct value.

### Validation of reference genes

The transcription factor gene *AGAMOUS* (*AG*), belongs to the MADS-box genes involved in the reproductive organ development in *Cycas edentata* [15]. AG was used to measure and normalize the most and least stable reference genes based off the analysis of geNorm, NormFinder and BestKeeper outputs. The experimental procedure for target genes was the same used for the selection of the reference genes. Samples from the megasporophyll, microsporophyll, female plant leaf, male plant leaf, asexual plant leaf, root, stalk and ovule were used to evaluate the relative expression level of the target gene, calculated using the 2^−ΔΔCt^ method and presented as fold changes [23].

## Results

### Identification and cloning of putative references genes in *C*. *elongata*

From the *C*. *elongata* transcriptome database, we retrieved thirteen putative reference genes using the sequences of *A*. *thaliana* orthologs as probes (Table 1). Since no *C*. *elongata* relevant genetic sequences information is available online, we cloned and sequenced the reference genes according to the selected sequences. Thirteen expected fragments of reference genes (sequences shown in S2 File) were successfully amplified with sizes ranging from 210 to 1248 bp (S2 Fig). Nucleotide similarity of *C*. *elongata* orthologs were confirmed by comparison to the [19] NCBI web site after sequencing. Our results showed that all expected fragments obtained from *C*. *elongate* have at least 74% similarity to those of *A*. *thaliana*, with *CeACT7* exhibiting the highest similarity (99%) with the ortholog of *Arabidopsis* (Table 1).

### Verification of primer specificity and efficiency

**Fig 2:**
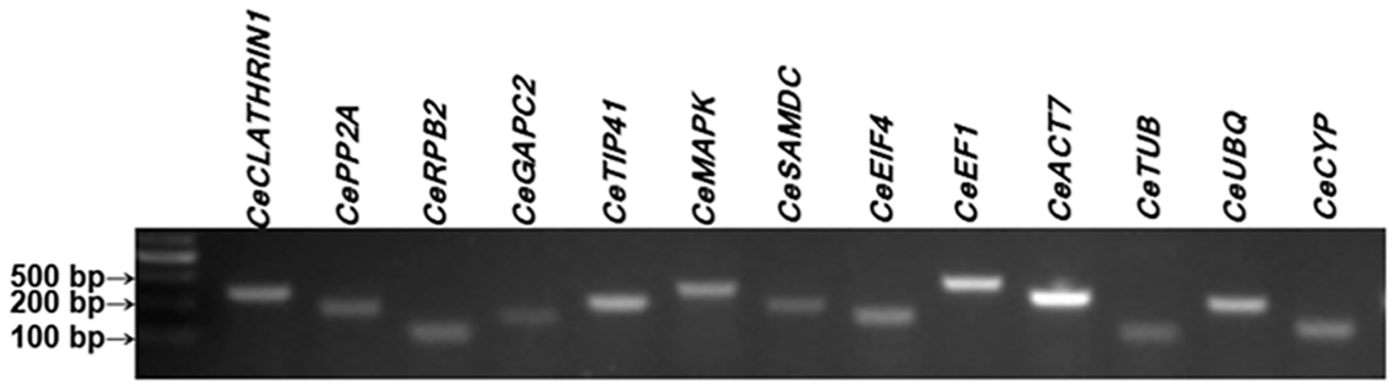
Agarose gel (2%) electrophoresis showing amplification of a specific qRT-PCR product of the expected size for each gene. 2000 bp DNA ladder marker was used.

Primer specificity was assessed through a confirmatory PCR and 2% agarose gel electrophoresis which revealed expected single bands of desired lengths (Fig 2). Furthermore, the specificity of primer pairs in qRT-PCR was also confirmed from a single peak in all melting curves and an absence of peak in the no template control (NTC) (S3 Fig). PCR efficiency (E) was estimated from the standard curve varying from 94.6% for *CeCYP* to 106.4% for *CeSAMDC* (Table 2, S1 File), which conforms to the acceptable range of PCR efficiency 90–110% [24] and correlation coefficients (R^2^) ranged between 0.990–0.996. Taken together, the results indicate that the designed primers could accurately amplify the 13 candidate reference genes.

**Table 2:** Candidate reference genes, specific qRT-PCR primers and different parameters derived from qRT-PCR analysis.

Gene	Primer sequences (5’ - 3’) forward / reverse	Tm (°C)	Aplicon Length (bp)	Primer efficiency (%)	R^2^
*CeCLATHRIN1*	CTTTTTGTTGGACGGGCTTT/AGGTGCTGTTGGTTGGCG	58	237	98.9	0.991
*CePP2A*	TGTCCAAAGATGGGGAGAGG/AGAGGGAATCATGAGAGCCG	58	184	102.3	0.992
*CeRPB2*	CGAGAAAGCATCCGCACAA/AAGAAACGCCAGCCAAATAAA	58	104	98	0.992
*CeGAPC2*	TCTGCCTCCTCGCCAATC/GCAAGCTGCACCACCAACT	58	153	104.1	0.99
*CeTIP41*	ACACATTCACAACACCATACCGT/CAGCCAACTCATCTTCATACAAAA	58	201	98.2	0.991
*CeMAPK*	CATCCGTGAGAGCCTGAGAAGA/GCCCACAACACCGAAACCA	58	253	105.4	0.99
*CeSAMDC*	CCTCCCAACCTTCATTTTCA/TGCATCAGGCAATCTTCTTG	56	193	106.4	0.99
*CeEIF4*	TCTCAGCTACCATGCCTCCT/CAGAGTGTGTCCAGCTTCCA	57	157	96.6	0.995
*CeEF1*	ATCATTCTTCCTCCTCGTTC/AGTGTCTCTTCTCCGTTTCG	57	287	97.4	0.996
*CeACT7*	GACATCTGAACCTTTCGGCA/GCTGGGCGTGACTTGACTGA	59	232	100.6	0.994
*CeTUB*	TGAGGCGAAGGATAAATGGTG/AATGCTGTTGGAGGCGGAACT	59	112	101.7	0.996
*CeUBQ*	AACTTTGGAGGTCGAGAGCA/GCCATCAACGAAGGTTCAAT	59	213	94.6	0.991
*CeCYP*	GCCCAAGGAGGGGACTTT/GCCCAAGGAGGGGACTTT	58	129	94.6	0.995

Primer locations in the coding regions of corresponding genes

### Expression profiling of reference genes

**Fig 3:**
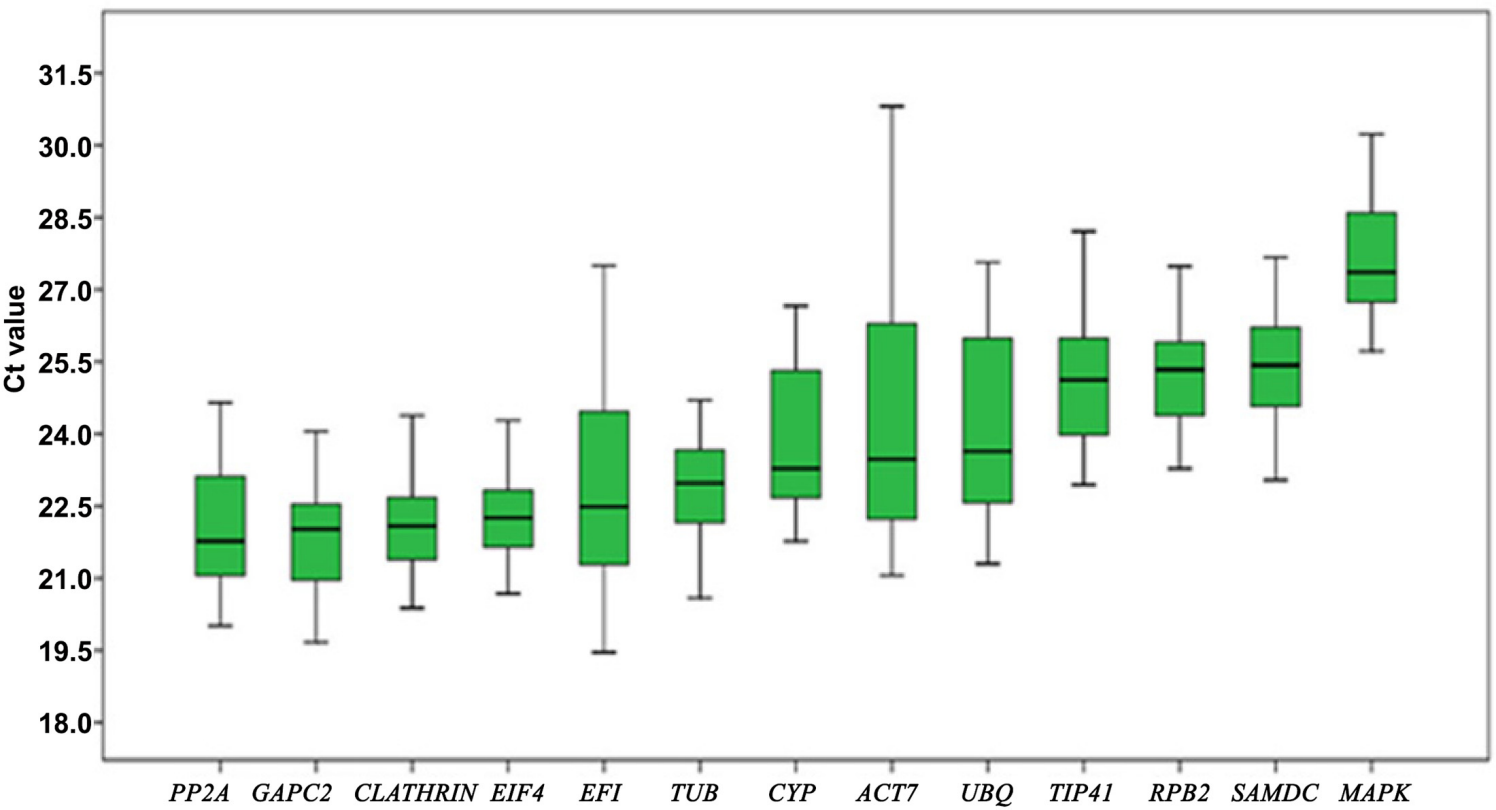
Box plot of the Ct value distribution of candidate reference genes in all *C*. *elongata* samples. The box indicates the 25th and 75th percentiles. Whiskers represent the maximum and minimum values; the thin line within the box marks the median.

Analysis of the Ct values (S1 Table) for each gene showed variation in the expression levels across all samples. Based on the Ct value interquartile range (25–75% percentiles) for each reference gene, the median Ct values ranged from 22 to 28 cycles (Fig 3), with *CePP2A* (21.77) being the most abundantly expressed gene followed by *CeGAPC2* (22.02), *CeCLATHRIN1* (22.09), *CeEIF4* (22.26), *CeEF1* (22.49), *CeTUB* (22.98), *CeCYP* (23.28), *CeACT7* (23.47), *CeUBQ* (23.64), *CeTIP41* (25.12), *CeRPB2* (25.34), *CeSAMDC* (25.43) and *CeMAPK* (27.36), the least expressed gene.

The lowest variation in gene expression was exhibited by *CeEIF4*, *CeCLATHRIN*, *CeRPB2*, *CeTUB*, *CeGAPC2*, *CeSAMDC* and *CeMAPK* with cycles below 2, while *CeACT7* with cycles above 4 depicted the most variation, depicted by larger box and whisker taps for *CeACT7* than for other genes (Fig 3).

### geNorm analysis

**Fig 4:**
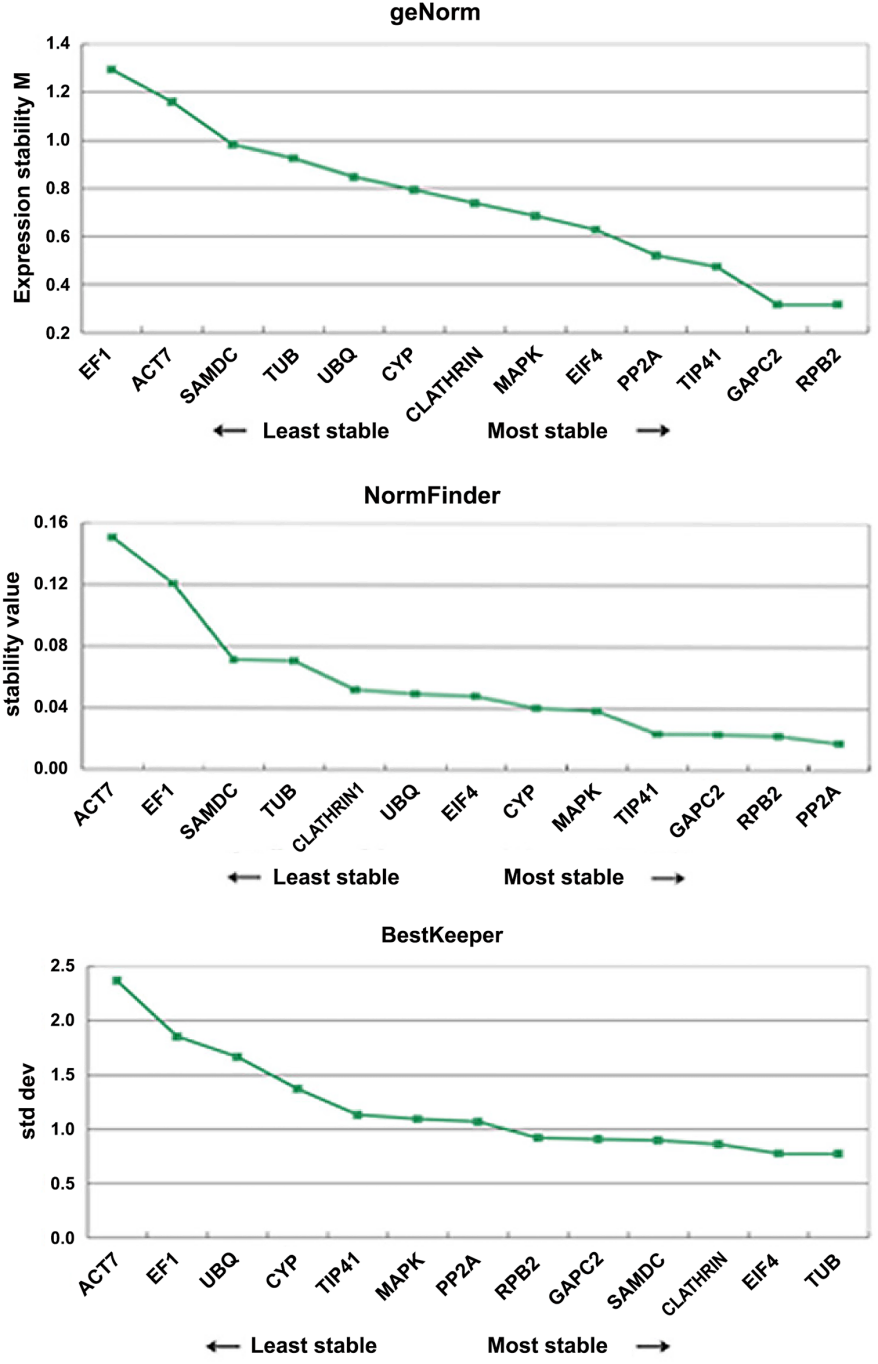
Expression stability and ranking of the candidate reference genes calculated by geNorm, NormFinder and BestKeeper. Ranking of 13 candidate reference genes (*CLATHRIN1*, *PP2A*, *ACT7*, *RPB2*, *EF1*, *GAPC2*, *SAMDC*, *TIP41*, *MAPK*, *CYP*, *UBQ*, *EIF4* and *TUB*) calculated by geNorm, NormFinder and BestKeeper methods in all samples (megasporophyll, microsporophyll, leaves of female plant, male plant, and asexual plant, root, stalk and ovule).

The program geNorm ranks reference genes based on the M value which is the mean pairwise expression ratio calculated from the average pairwise variation between a gene and all other control genes. It has been shown that M value and gene stability have a negative correlation [25]. geNorm recommends selecting reference genes with an M value below 1.5, which is supported by Vandesompele et al. who also suggest using M values lower than 1.0 to ensure the selection of the most stable gene[20]. Furthermore, M values below 0.5 indicate good stability of expression measure [25, 26]. In our analysis, 11 genes had M values below 1.0, and *CeGAPC2* and *CeRPB2* both had M values of 0.32, showing the highest expression stability, followed by *CeTIP41* with 0.47, *CePP2A* with 0.52, *CeEIF4* with 0.63 and *CeMAPK* with 0.69 (Fig 4, S3 Table). However, across all samples, *CeACT7* and *CeEF1*, the traditional control genes, showed the lowest expression stability (M value up 1.0). For the three subsets, *CeRPB2*, *CeGAPC2* and *CeTIP41* are the most stable genes in reproductive tissues, vegetative tissues, and leaves tissues respectively as ranked by geNorm (Table 3).

**Table 3:** Ranking of candidate reference genes in three subsets of *C*. *elongata* samples based on geNorm.

Reproductive tissues		Vegetative tissues		Leaves tissue	
geNorm		geNorm		geNorm	
Gene	M	Gene	M	Gene	M
*RPB2*	0.13	*GAPC2*	0.38	*TIP41*	0.22
*GAPC2*	0.13	*RPB2*	0.38	*RPB2*	0.22
*EIF4*	0.17	*TIP41*	0.39	*GAPC2*	0.29
*PP2A*	0.20	*CLATHRIN1*	0.49	*CLATHRIN1*	0.35
*CYP*	0.28	*PP2A*	0.56	*ACT7*	0.40
*TUB*	0.36	*MAPK*	0.61	*CYP*	0.46
*TIP41*	0.41	*EIF4*	0.67	*MAPK*	0.52
*UBQ*	0.46	*UBQ*	0.75	*EIF4*	0.56
*SAMDC*	0.51	*CYP*	0.82	*SAMDC*	0.61
*MAPK*	0.57	*TUB*	0.94	*PP2A*	0.66
*CLATHRIN1*	0.67	*SAMDC*	1.03	*TUB*	0.71
*EF1*	0.76	*ACT7*	1.17	*UBQ*	0.79
*ACT7*	0.93	*EF1*	1.35	*EF1*	0.88

**Fig 5:**
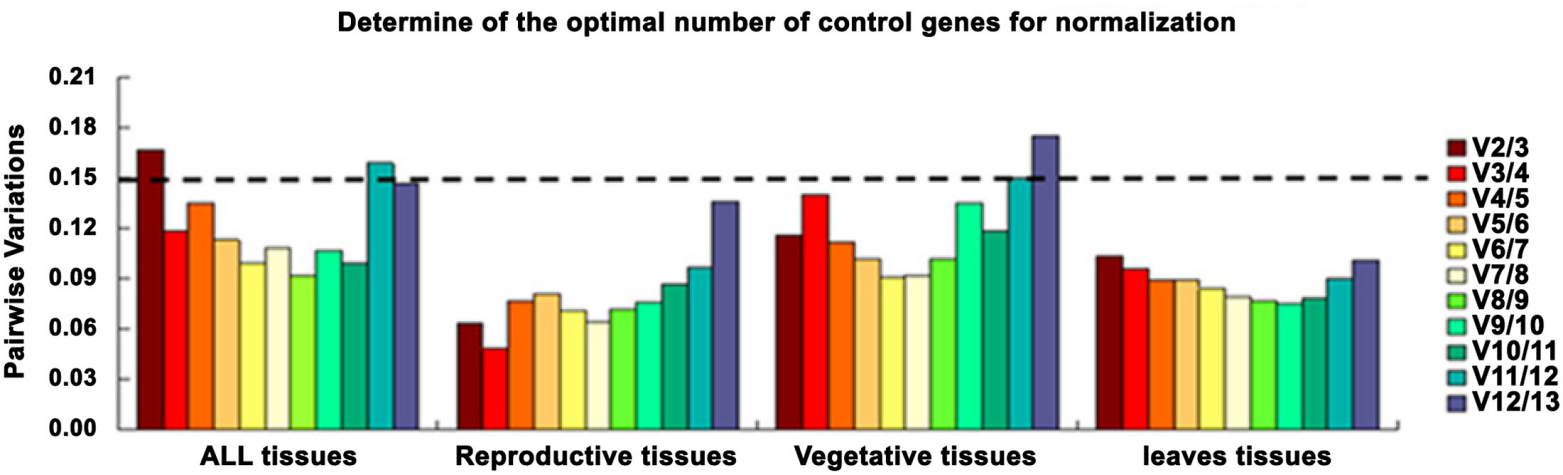
Pairwise variations (V) calculated by geNorm to determine the minimum number of reference genes for accurate normalization in four sets. The cut off value is 0.15, below which the inclusion of an additional reference gene is not required. All tissues (megasporophyll, microsporophyll, leaves of female plant, male plant, and asexual plant, root, stalk and ovule). Reproductive tissues (megasporophyll, microsporophyll and ovule). Vegetative tissues (leaves of female plant, male plant, and asexual plant, root and stalk). Leaves tissues (leaves of female plant, male plant, and asexual plant).

The optimal numbers of reference genes were calculated by pairwise variation (V_n/n+1_) [20] for data normalization. A threshold of 0.15 was set as the cut-off value for V, below which no additional genes were included. A result of V3/4 < 0.15 indicated that the inclusion of a third reference gene (*i*.*e*. *CeGAPC2*, *CeRPB2* and *CeTIP41*) was sufficient for an accurate normalization in total samples (Fig 5). When considering reproductive tissues (megasporophyll, microsporophyll and ovule), vegetative tissues (leaves of female plant, male plant and asexual plant, roots and stalks) and leaves tissues (leaves of female plant, male plant, and asexual plant) the calculated V2/3 was < 0.15 indicating that two reference genes would be useful for normalizing gene expression.

### NormFinder analysis

The NormFinder software can estimate the stability values of single candidate reference genes by calculating the intra-group and inter-group expression variability, with more stable gene expressions showing lower stability values. In total tissues, NormFinder ranked *CePP2A*, *CeRPB2*, *CeGAPC2* and *CeTIP41* among the six most stable reference genes, and *CeACT7* and *CeEF1* as the least stable control genes (Fig 4, S3 Table). The exception, *CePP2A*, was ranked fourth in gene stability by geNorm first by NormFinder. These discrepancies could be explained due to inter-group expression variations detected by NormFinder analysis, which is not taken into account by the geNorm algorithm [27].

### BestKeeper analysis

BestKeeper ranks reference genes based on the standard deviation (SD) and the coefficient of variation of their Ct values, where lower SD of Ct values indicate less variable expression compared to higher SD values. Genes with SD greater than 1.0 are considered unstable and should be avoided. *CeTUB*, *CeEIF4*, *CeCLATHRIN1*, *CeSAMDC*, *CeGAPC2* and *CeRPB2* (SD of 0.77, 0.78, 0.86, 0.90, 0.91 and 0.92 respectively) possessed SD values below 1.0, indicating low variation in gene expression, while *CeACT7* with SD of 2.48 and *CeEF1* with SD of 2.31 were the most variable in expression across all samples (Fig 4, Table 4). BestKeeper ranked *CeGAPC2* and *CeRPB2* as acceptable reference genes, corroborating the results from geNorm and NormFinder.

**Table 4:** Descriptive statistics and expression level obtained from BestKeeper.

	*CLATHRIN1*	*PP2A*	*ACT7*	*RPB2*	*EF1*	*GAPC2*	*SAMDC*	*TIP41*	*MAPK*	*CYP*	*UBQ*	*EIF4*	*TUB*
N	48	48	48	48	48	48	48	48	48	48	48	48	48
GM [CP]	22.03	22.03	24.28	25.18	22.85	21.73	25.41	24.99	27.58	23.82	24.05	22.26	22.87
AM [CP]	22.06	22.07	24.43	25.21	22.95	21.76	25.44	25.03	27.61	23.87	24.12	22.28	22.89
Min [CP]	20.38	20.01	21.06	23.28	19.46	19.67	23.04	22.94	25.72	21.77	21.30	20.68	20.59
Max [CP]	24.38	24.65	30.81	27.48	27.50	24.05	27.67	28.21	30.23	26.66	27.57	24.28	24.70
**SD [± CP]**	**0.86**	**1.07**	**2.37**	**0.92**	**1.85**	**0.91**	**0.90**	**1.13**	**1.09**	**1.37**	**1.67**	**0.78**	**0.77**
**CV [% CP]**	**3.90**	**4.84**	**9.69**	**3.65**	**8.08**	**4.16**	**3.52**	**4.52**	**3.96**	**5.75**	**6.91**	**3.48**	**3.38**
Min [x-fold]	-3.15	-4.06	-9.30	-3.74	-10.47	-4.18	-5.18	-4.14	-3.62	-4.14	-6.73	-3.00	-4.87
Max [x-fold]	5.09	6.14	92.61	4.91	25.14	4.99	4.78	9.33	6.30	7.16	11.47	4.05	3.55
SD [± x-fold]	1.82	2.10	5.16	1.89	3.62	1.87	1.86	2.19	2.13	2.59	3.17	1.71	1.71

N: number of samples; CP: crossing point; GM [CP]: geometric CP mean; AM [CP]:arithmetic CP mean; Min [CP] and Max [CP]: CP threshold values; SD [±CP]: CP standard deviation; CV [%CP]: variance coefficient expressed as percentage of CP level; Min [x-fold] and Max [x-fold]: threshold expression levels expressed as absolute x-fold over- or under-regulation coefficient; SD [±x-fold]: standard deviation of absolute regulation coefficient. SD and CV are indicated in bold.

### Validation of reference genes

**Fig 6:**
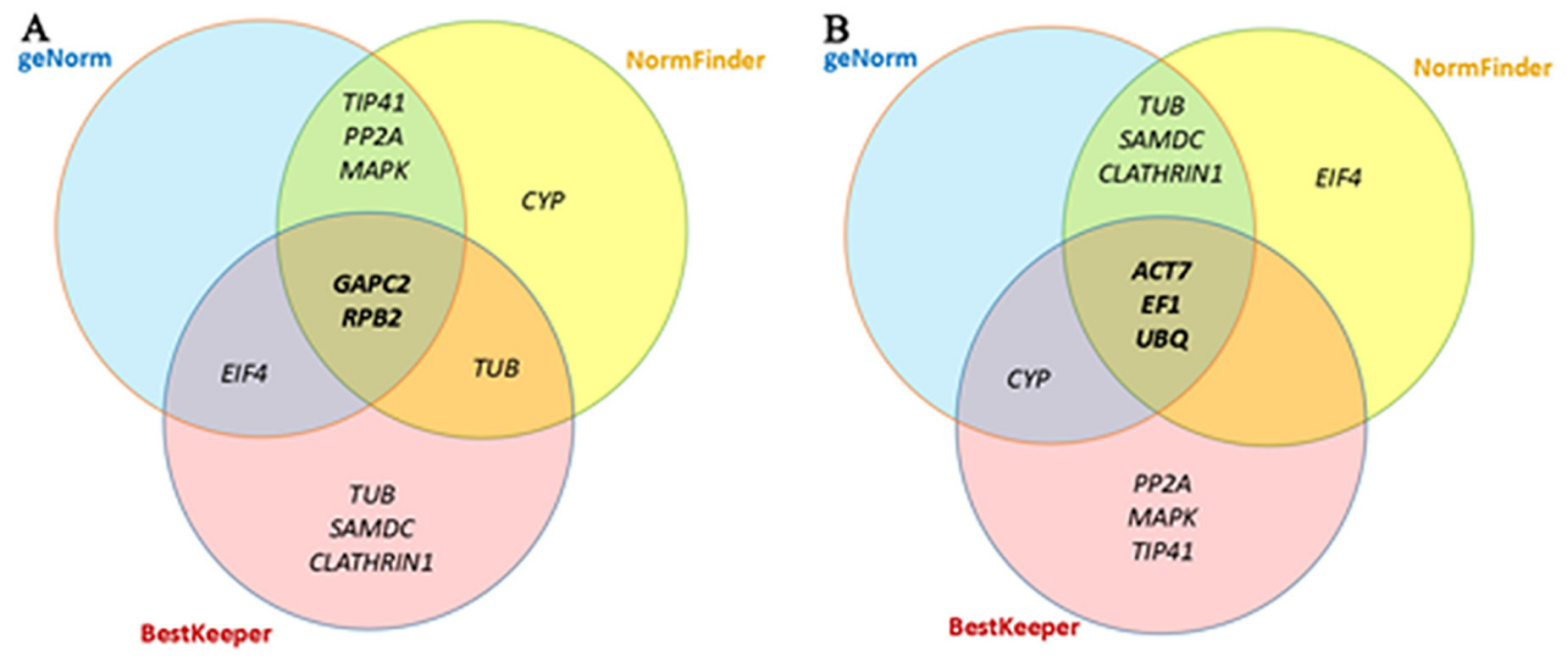
Venn diagrams. (A) The most stable reference genes present in the first six positions and (B) the least stable genes present in the last seven positions identified by the NormFinder, BestKeeper and geNorm methods. Diagrams were performed with the Venny 2.0.2 (http://bioinfogp.cnb.csic.es/tools/venny/index.html).

**Fig 7:**
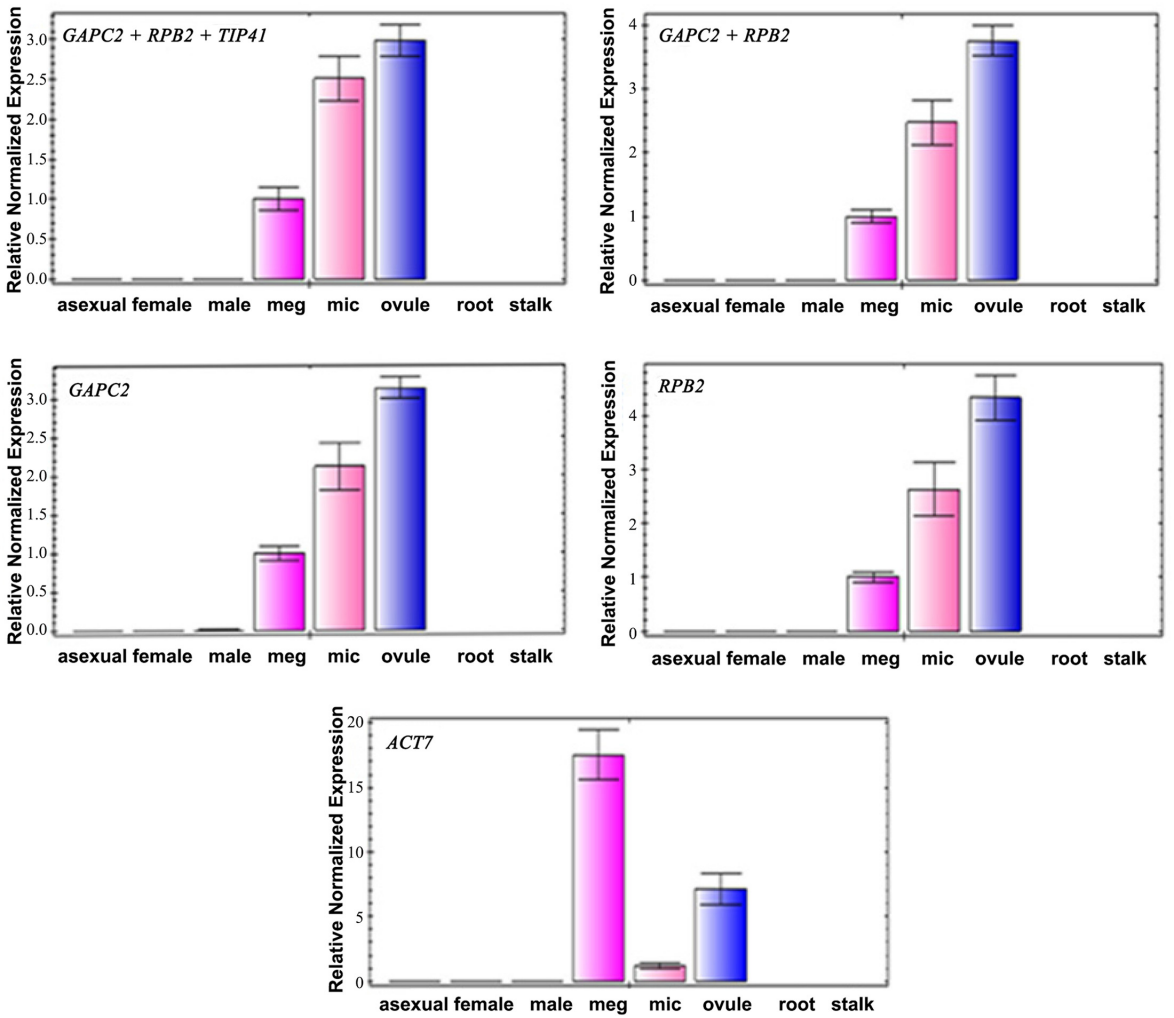
Expression levels of the *CeAG* gene. The most stable genes of different combinations (*GAPC2 +RPB2 +TIP41*, *GAPC2 +RPB2*, *GAPC2* and *RPB2*) and the least stable gene *ACT7* were used for normalization. The results presented are fold changes in relative expression compared to the meg tissue. The data are mean ± SD from three biological replicates. Samples were collected from asexual plant leaf (asexual), female plant leaf (female), male plant leaf (male), megasporophyll (meg), microsporophyll (mic), ovule, root and stalk of *C*. *elongata*.

To confirm the reliability of potential reference genes recommended by the combined analyses of three programs (Fig 6) while considering the optimal number of reference genes suggested by geNorm (Fig 5), four combinations of most stable housekeeping genes: *APC2 +RPB2 +TIP41*, *GAPC2 +RPB2*, *GAPC2* and *RPB2*, as well as the least stable gene *ACT7* were used in the normalization of the target gene *C*. *elongata AGAMOUSE* (*CeAG*). *CeAG* was identified and cloned (sequence showed in S2 File) utilizing the same processes described for reference genes. The Blastn showed that this gene shared 99% similarity to the genes of *Cycas pranburiensis* (KP238764), *C*. *elephantipes* (KP238762), *C*. *taitungensis* (KP238765) and *CyAG* of *C*. *edentata* (AF492455). The relative expression profiles of the target gene were performed in all samples and demonstrated that regardless of combinations of reference genes used, *GAPC2 +RPB2 +TIP41*, *GAPC2 +RPB2*, *GAPC2* or *RPB2*, a similar expression patterns of the target gene were obtained. The expression level of *CeAG* in different samples showed as: ovule > microsporophyll > megasporophyll, and no transcript level was observed in vegetative tissues (female plant leaves, male plant leaves, asexual plant leaves, root and stalk). By contrast, the relative expression of target gene with *ACT* as reference gene showed a more variable expression pattern, where the transcript level was as follows: megasporophyll > ovule > microsporophyll (Fig 7).

## Discussion

In plant molecular biological research, qRT-PCR is the most popular method of gene expression analysis and must be preceded by validated normalization to prevent non-biological event variations. This is particularly challenging for non-model species that do not have a reference genome sequence [5, 28–30]. The *C*. *elongata* is an example of a non-model species which is a gymnosperm in the family Cycadaceae. Cycads are considered the earliest separating lineage in extant gymnosperms for which is considered helpful to study the origin and evolution of MADS-box floral organ identity genes. Thus, the identification of suitable reference genes for *C*. *elongata* would be of considerable value [15, 31].

To our knowledge, this is the first survey of cloning and expression stability of qRT-PCR reference genes in *C*. *elongata* tissues. In our study, thirteen candidate reference genes were successfully cloned and identified, and their consistency of expression was further accessed using three statistical algorithms geNorm, NormFinder and BestKeeper in eight *C*. *elongata* tissues. When compared, the results of these three software programs were consistent although some inconsistencies were observed between these methods [32–35], which were due to the different algorithms used by the programs. For all methods, the *CeGAPC2* and *CeRPB2* genes were consistently selected as more stable whereas *CeACT7*, *CeEF1* and *CeUBQ* showed the least expression stability.

Similar to our selected control genes, analogous results were observed in *Gossypium raimondii* [18] for which *GAPC2* showed relatively stable expression levels in all tissues (*i*.*e*. leaves, shoots, buds and sepals). In previous studies, also found that, *GAPC2* was identified as a stable gene for normalization in citrus [26], different coffee cultivars [36] and *Brachypodium* [37]. For other studies, *GAPDH* was identified as the most variable reference genes in petunia when assessed during leaf and flower development [38] and in *Withania somnifera* [35].

Elements of our study were verified by previous work which showed that *RPB2*, a subunit of RNA polymerase II needed for elongation and mRNA transcription in eukaryotes [39] exhibited the best stable reference gene in *Plukenetia volubilis* [40] for seed development, and in citrus for leaf samples of different citrus genotypes [41] and litchi in different tissues [42]. However, *RPII* was classified as the least reliable references in coffee in nitrogen starvation, salt and heat stress [43].

Although *ACT* is one of the most commonly used reference gene in plants, in *C*. *elongata* it ranked last indicating low stability across different tissue samples whichever statistical methods assessed. Expression instability was also described by Czechowski et al. [7], who found *ACT2* to have the least stable gene expression among the 27 tested. Similar results were also observed in *Nicotiana tabacum* with viral infections [44].

We note that *EF-1α* has been considered a consistent reference gene and ranked as highly effective for use in gene expression studies with teak [27], *Picea abies* and *Pinus pinaster* [45]. While we report contrary results, our results corroborate those obtained by Xia et al. and Expósito-Rodríguez et al., which considered *EF1-1α* the most unstable gene showing low stability in African oil palm and in tomato [8, 46]. In accordance with similar studies of gene expression in teak [27], brazilian pine [5] and rubber tree [47], the reliable reference genes for *C*. *elongata* also varied within subsets of samples, suggesting that the most constant expression profile varied between tissues and/or samples even within the same plants. Taken together, these results suggested that reference genes may show a different stability pattern under different conditions or in different plants even within the same plants, which suggests reliable reference genes are highly specific for a particular experimental setting. This argues that a careful evaluation for every individual experimental setup is indeed needed.

It has been reported that the added reference gene has a significant effect on normalization and should be included in calculations [20]. From our results we found that the inclusion of a third reference gene (*i*. *e*. *GAPC2*, *RPB2* and *TIP41*) when considering the total samples was an improvement. The optimal number of internal control genes is frequently confirmed by the threshold ≤ 0.15 [20], nevertheless this is not absolute since small datasets require fewer reference genes than larger ones and previous studies have reported proper normalization with higher cut-off values [27,44]. Therefore our results point out that V2/3 for all tissues is 0.167 which is close to 0.15 and considering the increased costs of more than three reference genes in large scale gene expression profiles, two housekeeping genes in *C*. *elongata* is recommended.

To illustrate the actual utility of validated reference genes in this study, the expression pattern of *CeAG* was examined in *C*. *elongata*. *AGAMOUS* (*AG*) is the class C MADS-box gene that have been shown to play key roles in the determination of reproductive floral organs such as stamens, carpels and ovules [48]. In Gymnosperms, *AGAMOUS* is also expressed only in the reproductive structures, which was reported in *C*. *edentata* [15] and *Ginkgo biloba* [49]. Our results demonstrate that the expression pattern of *CeAG* were comparable across the different internal control combintations tested (*GAPC2 +RPB2 +TIP41*, *GAPC2 +RPB2*, *GAPC2* or *RPB2*), which suggested that the relative expression of the target gene was highest in ovule, followed by microsporophyll, megasporophyll and expression less in vegetative tissues (Fig 7). This shows that the use of three or more reference genes is unnecessary supporting our recommendation for the optimal number of control genes. This expression pattern is consistent with the results obtained by Zhang et al. [15] that showed *CyAG* can only be detected in reproductive tissues of *C*. *edentate*. Similarly, *GBM5* had a higher expression in ovules than stamens of *Ginkgo biloba* [49], which confirmed our selection of best stable reference genes. On the other hand, when the most unstable *ACT7* was used as reference gene, the expression pattern of the target gene showed a distinct trend toward the highest transcript level in megasporophyll, where it displayed the lowest expression level when appropriate reference genes were used. This indicates that the use of an inappropriate reference genes may cause erroneous results leading us to conclude that, the *ACT7* gene is not appropriate for gene expression studies in *C*. *elongata*.

In conclusion, the proposed reference genes *GAPC2* and *RPB2* are suitable for qRT-PCR normalization in different tissues of *C*. *elongata*. To our knowledge, this work represents the first attempt to clone, sequence and evaluate commonly used candidate reference genes in *C*. *elongata* for the normalization of gene expression analysis using qRT-PCR. It will facilitate further gene expression analyses of target genes in different tissues of *C*. *elongata*.

## Supporting Information

S1 Fig Total RNA from *C. elongata* analyzed by agarose gel electrophoresis. (DOC)

S2 Fig Agarose gel (2%) electrophoresis showing amplification of a specific PCR product of the expected size for each gene. (DOC)

S3 Fig Specificity of qRT-PCR amplification. (DOC)

S1 File Standard curves of all the primer pairs in this study. (DOC)

S2 File *Cycas elongata* cloned sequences of *CeAG* and 13 reference genes. (DOC)

S1 Table 260/280 ratios and 260/230 ratios of all samples analyzed by ultraviolet spectrophotometer. (DOC)

S2 Table Raw Ct data used for statistical analysis in this study. (DOC)

S3 Table Ranking of *Cycas elongata* reference genes calculated by geNorm, NormFinder and BestKeeper. (DOC)
