# Supplementary figures and images for: Selection and Validation of Reference Genes for qRT-PCR in Cycas elongata
Source: PLoS One. 2016 Apr 28;11(4):e0154384. doi: 10.1371/journal.pone.0154384 (PMC4849791; doi:10.1371/journal.pone.0154384)

S1 Figure. Total RNA from C. elongata analyzed by agarose gel electrophoresis.

**
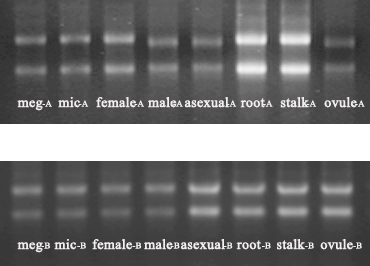
**

Supplement: S1 Fig — (DOC) [file pone.0154384.s001.doc]
