# Supplementary material for: Selection and Validation of Reference Genes for qRT-PCR in Cycas elongata
Source: PLoS One. 2016 Apr 28;11(4):e0154384. doi: 10.1371/journal.pone.0154384 (PMC4849791; doi:10.1371/journal.pone.0154384)
Supplement: S2 Fig — (DOC) [file pone.0154384.s002.doc]

S2 Figure. Agarose gel (2%) electrophoresis showing amplification of a specific PCR product of the expected size for each gene.


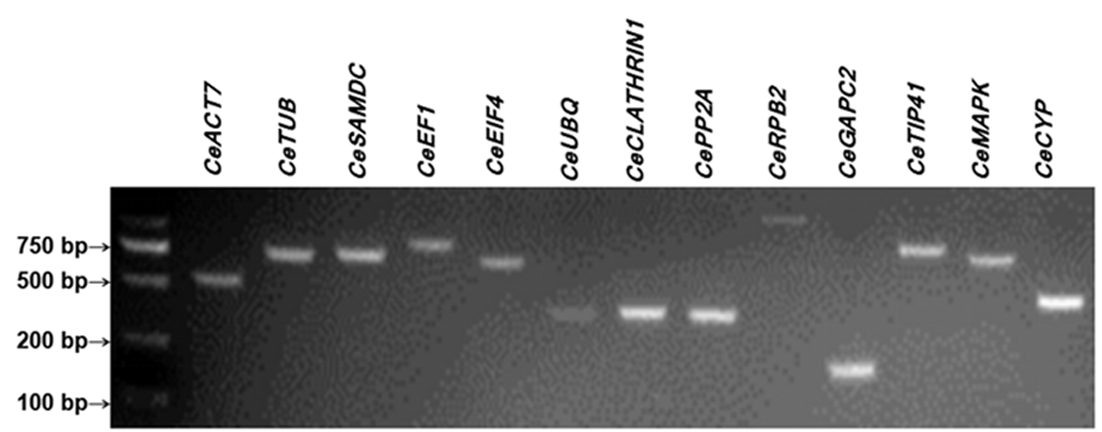


2000 bp DNA ladder marker was used.
