# Supplementary material for: Selection and Validation of Reference Genes for qRT-PCR in Cycas elongata
Source: PLoS One. 2016 Apr 28;11(4):e0154384. doi: 10.1371/journal.pone.0154384 (PMC4849791; doi:10.1371/journal.pone.0154384)
Supplement: S3 Fig — (DOC) [file pone.0154384.s003.doc]

S3 Figure. Specificity of qRT-PCR amplification.


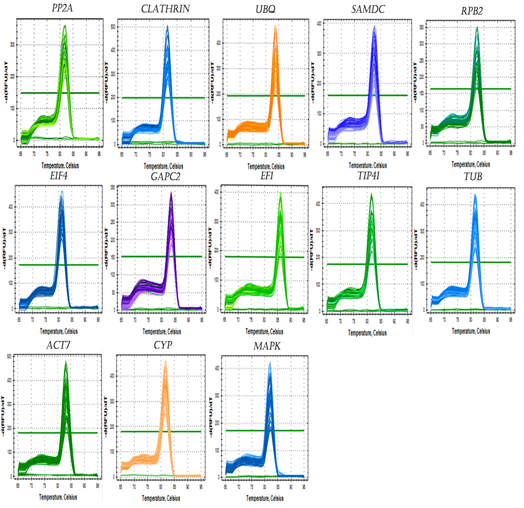


Melting curves (dissociation curves) of the 13 amplicons of candidate reference genes after the qRT-PCR reactions, all showing one peak.
