# Supplementary material for: Selection and Validation of Reference Genes for qRT-PCR in Cycas elongata
Source: PLoS One. 2016 Apr 28;11(4):e0154384. doi: 10.1371/journal.pone.0154384 (PMC4849791; doi:10.1371/journal.pone.0154384)

S1 File. Standard curves of all the primer pairs in this study.

*CLATHRIN1*

*
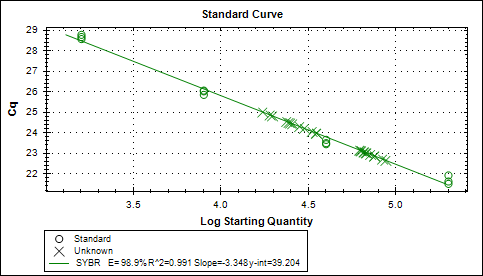
*

*PP2A*

*
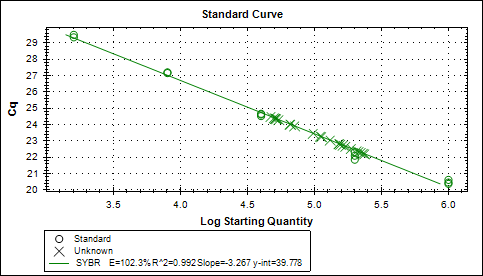
*

*RPB2*

*
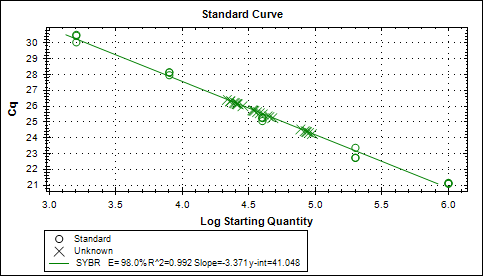
*

*GAPC2*

*
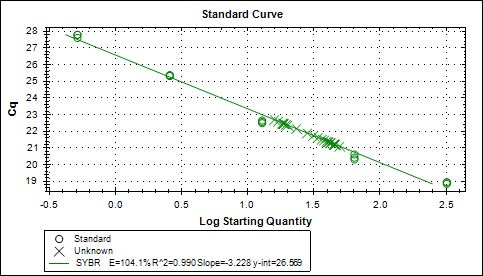
*

*TIP41*

*
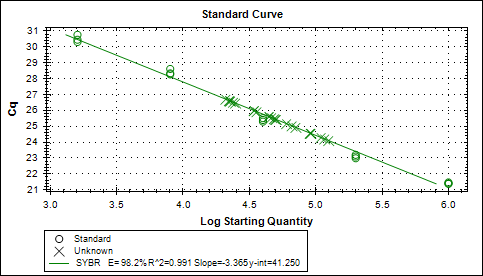
*

*MAPK*

*
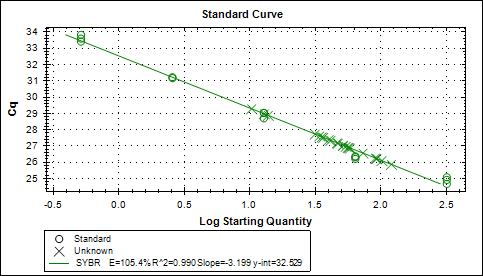
*

*SAMDC*


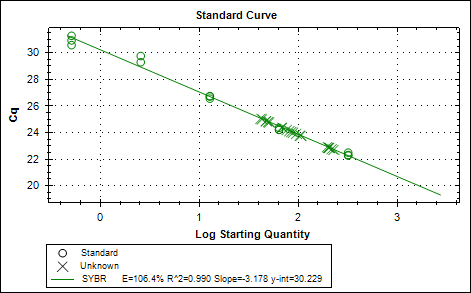


*EIF4*

*
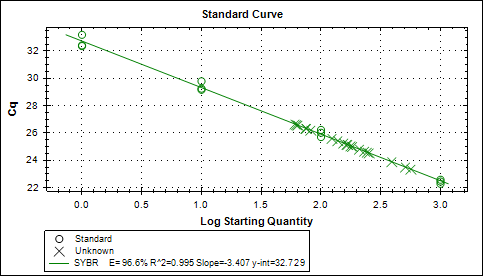
*

*EF1*

*
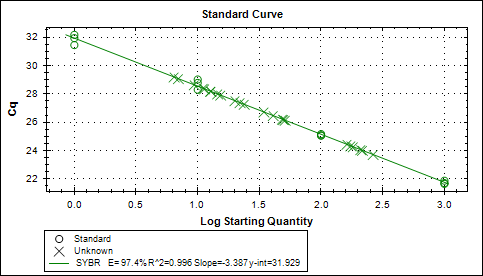
*

*ACT7*

*
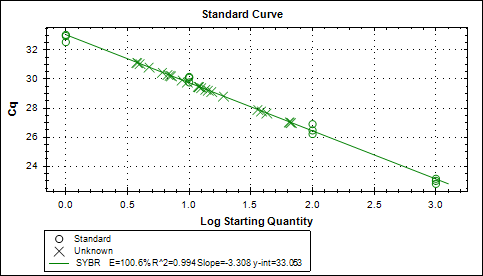
*

*TUB*

*
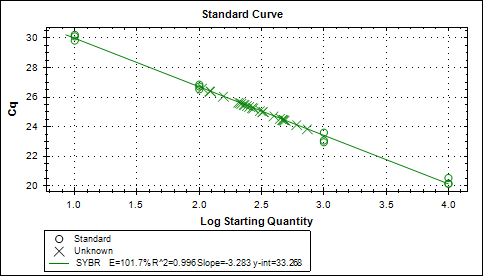
*

*UBQ*

*
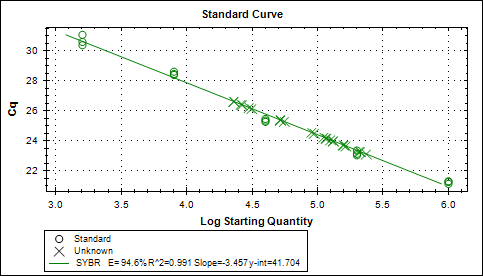
*

*CYP*

*
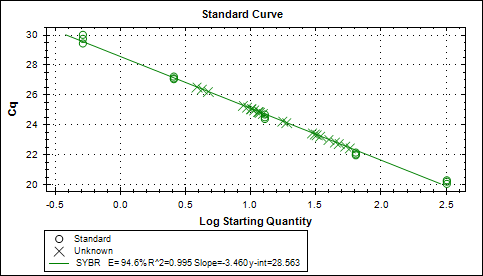
*

*CeAG*


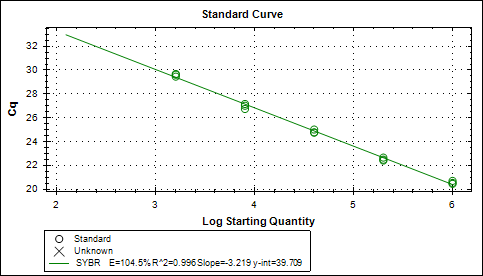

Supplement: S1 File — (DOC) [file pone.0154384.s004.doc]
