# Supplementary material for: Selection and Validation of Reference Genes for qRT-PCR in Cycas elongata
Source: PLoS One. 2016 Apr 28;11(4):e0154384. doi: 10.1371/journal.pone.0154384 (PMC4849791; doi:10.1371/journal.pone.0154384)
Supplement: S2 File — (DOC) [file pone.0154384.s005.doc]

S2 File.*Cycas elongata* cloned sequences of *CeAG* and 13 reference genes.

>*Cycas elongata MADS-box transcription factor AGAMOUS (AG)*

GCGTGTAATGATGAGCTGCGGCCTCGATTAGATTTGCATGAAGAAAGTTTCGAGAATCAAATGCAGGCAAGGCATCAAATTCAGGTCCCGGGATTAGGACATTTGTATTCTGGTTGCTTTCATATTCCGCTATCTTAGTGCGAAGAAACTGATTCTCGGCTAGTAATATGTGTTCCCTTCTTTGCATAATCTCGATCTCTTCGAGCAGCATTTCATTCTTCTTTGATCGTACTCGGCTGAGGCCTCTTTCTAGTCGAATTTCAAGCTGCTTAAGTTCCTTTACGCTTAAAGATGTAAGTGCATCTCCCATTAGGTGTCTGTTAGCATTTTGTAGAATGTCAATCTGCTGCCTGAGTTTTCCTGCCTCCTGTTGCCAGTACTGAGAATTGGACTCTGAAATGGCTCCTCCCCGCGTGTTGTCAGCGCCAGTCTTT

>*Cycas elongata Clathrin Adaptor Complex*

CTTCACCACTACTCCCAATGCAAACATTTTTGCACCAAACACGCTCTTTACCTTCACATTCACTTCCATACGTGTCCGACCCAGTTCCTTAATGCTTGGTAGAACCCGAAATGGAAGGTTAACTCCTTCGGTAATACGATACTTCATCAGTTCAAATTCTCCATCAGGTGGTACAAAACTAACTGTCTTCTCTGAGTTGAACCGTGTCAAGTTTACACACTGATGGAAAGTGACATCATCAAGCTCAATTGTCTTTCCGCTTTTTGTTGGACGGGCTTTAATCTGCGATTCCTTTTCAAGCCCTATTTTATCATTCAAGCCTAACTTTAAGTCAGGCATGCCTGAAAGAAAACATTTCATAAGAATCTTCCCAGTAACGTCACAGCGAAGGACACTCCCTTTTGAAGACATCAGG

>*Cycas elongata serine/threonine-protein phosphatase PP2A-1*

GTTATCAAGCGTGTCCAAAGATGGGGAGAGGCCACCATGCAGGCAGAAGATCTGACTTTCAATGAGTGCAGTCAAAGGCAAGTAGTCAAAGAGGTCTGTAAAATCCTTCCACACATTTGCATTGCCATATTTTCTTAGGCACTCATCGTAGAACCCATACACTTGTGTAATCTGCCGGCTCTCATGATTCCCTCTTAGTATAGTAATTCTGTCTCTGTAGCGCACTTTCAAGGCAACTAGAAGCGTCACCGTCTCAACAGAATAGTATCCACGGTCTACATAATCGCCCATGAACAGATAATTAGTGTCGGGAGCCTTGCCACCGATGCGAAACAACTCGATCAAATCATGGAACTGCCCGTGAATGTCTCCGCATACCGTGACCGGGCATTTCACAGGTTGCACATTC

>*Cycas elongata RNA polymerase II subunit RPB2*

TCTTTTGCAGGTAAGACCTCACATCCCTTGTCAGTCTGCGAAAGAGCCCTCGGAATAGTCCTCCAAGTAAAGGACCAGCAAGATCCAATCGTTTGTTACCATAGTGATCTCTATCATCTTCCGGCCGTCGATCAAGTGCACACATCAGAAGCCGATGGATGATGTATCCAAAATAATAAGCTTTCTTTGTTTCGCAACCCTCTCCAATCTCAACATGAGGCAGCATTTCCTTCTGTAAAATTTCTTTAGCATACTTTATACGTTTTTCTCTTGTAACGCCAACAGTTGATCCACGTTTCCCAATGTAATCCAGAGCCACCTGTTGATTCTGAATAACAAATGCTTCCTCCAATGATGGCCTCAGCAGTTCCATCATTGCAGCATCATTAAAATCATAGCATATATGTTCTAATATGTCTTTATCAGCAACAAATCCCAGTGCTCGAAACACAATGATAATTGGAATTTCAGAGCGAATATATGGTAGAGTGGCTCGAATATACTGTCCTGATGAACCCCCTTTGGCACTTGCTCGAGAAAGCATCCGCACAAACATACTGCTTGGTGGCCTGTTCTGTGCTTCAGCCATCGATCTGACCTCCGCAGCATAAGAATATTTATTTGGCTGGCGTTTCTTGAAAACATAAACATGGTTGGTACTCATTTTTTCTTGAGCAATGAGAACCTTCTCACTTCCATTGATAATGAAGTATCCACCTTGGTCATATGGGCACTCTCCAAGCTCAGTGAGATCCTTCTCAGAATTCTGATACAGGGTGCAGTAACTAGACCGCAGCATTATTGGTACCTTCCCGATGAAAACTTTAGTAAGATCTTGTTTTTCTGCTAGCTCTTCACAGTCAGGTCCTTTCTTGTAAACACATTTTGTCACATCAACATATAGTGGAGCTGAGTAGGTCAAATTTCTTAATCTTGCAGCCTTGGGAAACAAAGTTGCAGTTTCCCCATCTGATTCTGTCATCATAGGTTTGCTCAGATATATTTGACCAAAGCTGATCTTGTAGACTGTCTCGACAAATTCAGCCTGCCTGCCAGGATTATGCTGCGCTTCCGGCCGAATCTCAATGTCGGCAGACTCGTCCACGATTTCCTGCATAGTGTTCTGAATGAATTCATCGAAGGAATCCAGTTGCTGCCTCACCAAACCCTTATCTTCAAAGTAGGCACTGATGACTGCCCAGGCGTCTTCCTGTGTAATTTCCTCATCCTCGTCCACATAATCTTCGT

>*Cycas elongata glyceraldehyde3-phosphate dehydrogenase GAPC2*

GGCCATTCCAGTCAATTTTCCATTGAGAGCCGGTAAAACCTTGCCAACAGCCTTGGCAGCTCCAGTACTGCTAGGAATAATGTTAAATCCAGCCCCTCTGCCTCCTCGCCAATCCTTGTTAGATGGTCCATCAACTGTCTTCTGAGTTGCTGTAATGGAATGAACAGTGGTCATGAGACCTTCAACGATGCCAAACCTTTCATGGATGAC

>*Cycas elongata TIP41-like protein tonoplast intrinsic protein4*

AGACAGCTCTCTTGAACTTGTACATGAGGAAACTGGAATTAAAATTCACTTTAATGCACTTGATGCTCTCCATGAATGGAAGCAAGAAGCTTTACCGCCTGTTGAAGTGCCAGCTGCAGCCAAGTGGAAATTTAGAAGTAAGCCAAGTCAACAGGTGATATTGGATTATGATTACACATTCACAACACCATACCGTGGCAGTGAAATAATTGAGCCAAACAGAAAGCAGAAAGAGATGGACGTTTCCCCAAAAGAAACAAATGAAAACTTGTGCTGGGAAGAGTGTGACGACCGCATCAATTTGGTCGCATTACAGTCAAAGGATCCAATACTGTTTTATGATGAGGTGGTTTTGTATGAAGATGAGTTGGCTGATAACGGTGTTTCGCTTTTAACAGTAAAAGTGAGAGTAATGCCAACTTGTTGGTTTCTTCTTTTGCGTTTCTGGCTGAGAGTTGATGGTGTACTCATGAGAATAAGAGACACTCGGGTGTATTGTGCTTTTGAAAGTGATGTCAAAAGGATACCTAAAGTTATTCGCGAGTGCTGTCGAAGAGAAGACACATTTCAGGCATTAGCCGCAAGGGGTTTTCCATCAGATCCTTCTGCATACAGTGATCCCAACTTAATTAGTGAAAGACTGCGAATCCATTGGCAAACTCATGAAAAGCTCAGTCTGAAGTAGAAATCATAGGTGGCATGAGAGTCCACTGTTTGATAACTAAAGTGAATATTGGATTGAGTCAACAATTTGCATTTGGCACTGGGTGCAAAATCCCAAGTCTGAATCTCATCCTGGATAAAAGAATAGAGAAAGCTGCTGCAGGGATTTGGCCAGATCGCATAGTTACTACTTGTATATTGAACGGTGGACTATGGTCCATTCTGGTTTGCATTTCA

>*Cycas elongata mitogen-activated protein kinase 1 MAPK*

GTGATTCTCTTACAGGGGTCAAAAACAAGCATTCTACTTAATAGATTGATTGCTGCCGCATTCGCATGAGAATATACATGCTCCAGAGACCTGCGAGGCGTGGCAGGAAGTGATTTTATGTAGCTGTAAGCTTTCTGGCTCTGAACGAAACCGAGATCACCTTCATCTGGGCTGCCAAGTACATTGATGATCAGCTTCAGTTGATTGATATAATCCGTACCTGGGAAAATGGGCTTTCGGCCTAGCAACTCCGCGAAGATACAGCCGACAGACCACATGTCAATGGATGTGTCGTAGTCATCACATGACAGAAGCAGTTCTGGAGCCCTGTACCATCGCGTGACAACATACTCGGTCATGAACATTCCTTTCTCGACAACAGTCCGAGCCAGACCAAAGTCACATATCTTAAGGTCACAACTGGCATTCAAGAGTAAATTACTGGGCTTCAGGTCTCGATGCAGAACATTTGCAGAATGCAAATACTTCAGGCCTCGAAGTAACTGATATATGAAATACTGGCAATGGTCATCCGTGAGAGCCTGAGAAGATCTGATAATTTGATGCAAATCCGTATCCATGAGCTCACAGACAAGATACACATCATTGAAAGTGAACCTTTCAACGGGCATCATGATATCTTTGACGGCAATTATATTATCATGGCGAAATTGGCGAAGAAGCCTGATTTCACGAAGGGTTCTCTGTGCATCTGTCCGATTCTCAAAGGCATTTGTGATCTTTTTAATGGCAATATTCTCGTTGGTTTCGGTGTTGTGGGCAGAACAGACGATACCGTATGA

>*Cycas elongata S-adenosylmethionine decarboxylase SAMDC*

AAACCTTAGAAGCCCACAATGATTCATTCTGTATGTATGATACACAACACTACTCTGACCTGGAAGCTCCTGATGGATTGTCCTCTCACACACATACCCCTGTGGCCAAACTGGCTTAGTCCAAGAAGATTGCCTGATGCAAGCATTCACATGAACTGCAACAGAGAAAACCTTAGGCTGGAAAGAACTTAATACTCTCTCCACAACTGCTTCAAAGTCACACTTCCTGGGATTATAACCCATTGCCTCGAAGCTTGCATAGCTAAACCCATCTTCAGGAGTTACATGAATGGTTGAAAGAGCTGATCCTTCAATTGCATTCATTGAGTACCCACATGGATCAAACTCAAAGTCACATATTTCTGAATCTGGAAGAATGTTGCATATTCCTGAATTTGCAGTCATTTCAGCTGCAGATTTAGAATTACCCTTGTAAAAGACAGATGCCTGTTTGCTGTCCAATTCAGTCATGCACATTTCCAATGTAAATGTTGGATCAATACAATCACCCCAAGTAGTCTGGTCAGTACAATCACCCCAAGTAGTCTGTTGAGCCAAGGCAGAATAAATGTGCCATTTATGAGGTATTCCATAATCCCCCAATACATAAGCCTTTCCACTCAAACCAAGCATTCCAAAATACTTCTCCAGGTAAGCGACTTCCTCAGCAAAACTGCGGTGAGGAAATGGTTGGGCCTGAGGGAAAATAAATGAGCCACGAGTGTACTTAACACA

>*Cycas elongata eukaryotic initiation factor 4A-3*

AACCTTCCGAGCTGTATTTTCTGTGTGGACTAACTGTTTTTGACTATACGGTAATTGCAGCCATGGCGACAGAGGGCACCCACTTCGATGCTCGTCAGTATGACGCCAAAATGAACGAAATCCTTTCAAACGAAGGCGATGAGTTCGAATCGCCCTATGAGGAGGTCCACGAGAGCTTTGACTCCATGGGCCTGCACGAAAATCTTCTCAGAGGCATATATGCTTACGGTTTTGAGAAACCCTCTGCCATCCAGCAGAGAGGAATTGTGCCCTTCTGTAAGGGACTTGATGTGATCCAGCAAGCTCAATCTGGAACCGGCAAGACTGCAACCTTTTGTTCGGGGATTCTGCAGCAACTGGATTACAACTTGCTGCAATGCCAGGCATTGGTTCTTGCTCCTACCCGAGAGCTTGCCCAACAGATTGAGAAGGTCATGCGGGCCCTTGGTGACTACTTGCAGGTCAAAGTCCATGCCTGTGTCGGAGGGACCAGTGTTCGTGAAGATCAACGCATTCTGATGAGTGGGGTGCATGTCGTCGTTGGAACGCCGGGTCGTGTGTATGACATGCTGCGCAGAAATTCTCTGCATCCAGATTACATGAAAATTTTTGTGTTGGATGAAGCCGATGAAATGCTTTCCAGAGGTTTCAAGGACCAGATTTATGATATTTTTCAGCTTCTTCCTCCAAAAATTCAGGTCGGTGTTTTCTCAGCTACCATGCCTCCTGAGGCTCTTGAGATCACTAGAAAGTTTATGAATAAGCCTGTAAGGATTCTGGTGAAGAGGGATGAGTTGACACTTGAGGGTATCAAACAGTTCTATGTTTATGTTGATAAGGAGGACTGGA

>*Cycas elongata elongation factor 1-alpha 2*

CAGCGAATTTTGAGAAGTGGTGGTTTTAGCAGAGAAGAAATGGCAATGGCGATGGGAATATCGATGGCTCCGACGGTTCCATCGTCGATGGCGAAATCCAAGGAATCATCACAAGGTACAGTCTCATTTGCTCTGAAACCCAAAACCTTGAGCTCATCCCTTGCATTTTCCAACAACATCAGTTGCCCCAAAGGTTTCAGAGATCATTCTTCCTCCTCGTTCAACACCGGCAGACCGTTCCGGTTCACTGTAAGAGCTGCCAGAGGAAAATTCGAGAGAAAGAAACCCCATCTGAACATCGGAACCATCGGTCATGTGGACCACGGGAAAACCACCCTCACGGCCGCCCTGACAATGGCCTTAGCCTACTTTGGAGGGAGCGCGCCCAAGAAATACGATGAAATCGATGCTGCACCAGAGGAAAGGGCCAGGGGAATTACCATCAACACTGCCACGGTGGAGTACGAAACGGAGAAGAGACACTATGCCCACGTGGATTGCCCCGGCCACGCGGACTATGTGAAGAATATGATCACCGGGGCGGCCCAAATGGACGGGGGTATCCTGGTCGTGTCCGGAGCGGACGGTCCTATGCCCCAGACCAAGGAGCACATCCTGCTGGCCAAGCAGGTGGGGGTGCCAAACATGGTGGTTTTCCTTAACAAACAGGACCAGGTGGACGACGACGAGCTGCTTCAGTTGGTAGAGCTCGAGGTCAGGGAGCTTCTGACTTCGTATGAGTTCC

>*Cycas elongata actin 7*

GGAGGTGCTACAACCTTTATCTTCATACTGCTTGGAGCAAGTGCAGTGATTTCCTTGCTCATGCGATCAGCAATTCCAGGGAACATAGTAGAACCGCCACTGAGTACAACGTTCCCATACAAGTCTTTACGAATGTCCACATCACACTTCATTATGGAGTTGTAGGTAGTCTCGTGGATACCAGCAGACTCCATTCCTATTAAAGATGGTTGGAACAACACCTCTGGACATCTGAACCTTTCGGCACCAATGGTTACTACCTGGCCATCAGGAAGTTCATAGTTCCTTTCTATAGCAGAGCTTGTCTTTGCAGTCTCCAACTCCTGTTCGAAGTCAAGGGCAACATAACCTAGCTTCTCCTTCATGTCTCTAACAATCTCCCGCTCTGCAGTTGTGGTGAAGGAGTAGCCACGTTCAGTGAGAATCTTCATTAATGAATCAGTCAAGTCACGCCCAGCAAGGTCCAGTCTGAGAATGGCATGAGGCAAAGCATACCCCTCATAAATTGGGACAGTGTGTGTAACTCCATCA

>*Cycas elongata tubulin alpha-6 chain*

GTTGGCCTCTCAATATCCAAGGATCGTCTGCAGATATCGTATATGGCTTCATTATCTAGCAACACTGCAACATCTGTGTGTTCTAGCAAAGAATGAGTTGAGAGGACGCTATTGTAAGGTTCCACGACCGCGGTAGAGACCTGAGGCGAAGGATAAATGGTGAATCCGAGCTTTGACTTCTTGCCATAGTCGACAGAAAGCCTCTCTAAAAGTAAGGAACCCAAACCAGATCCAGTTCCGCCTCCAACAGCATTGAAAACCAAAAATCCTTGTAAACCCGTGCAGTTGTCTGCGAGCTTCCTCACCCGATCCAGACAAAGGTCAACAATCTCCTTGCCCACCGTGTAATGACCGCGAGCGAAATTATTGGCGGCATCCTCCCTGCCGGAGATCAACTGTTCAGGATGGAAGAACTGCCGGTATGTTCCGGTCCGGACCTCGTCGGTGACCGTGGGCTCTAGATCTACAAATATGGCCCTTGGCACATGCTTCCCCGCCCCCGTCTCGCTGAAAAATGTATTGAACGCATCGTGCGCCACACCCACGGACGCATCACTGGGCATCGTCCCGTCGGGGTGAATTCCATGCTCCAGACAGTAAAGCTCCCAGCACGAATTTCCCACTTGAATCCCCGCTTGGCCGATGTGAATACTGATAATTTCTCTCATTTCCTTTCCAAGCGACTGATTGAGATTGAGAATTCCAGCACAGATGAGGTCTGAAGAGGGATT

>*Cycas elongata ubiquitin 11*

GACGGGAAAGACCATAACTTTGGAGGTCGAGAGCAGCGACACCATCGATAATGTCAAGGCTAAAATCCAGGACAAGGAAGGGATCCCCCCCGATCAACAACGGTTAATATTTGCGGGCAAACAGTTGGAAGACGGTCGCACTTTGGCTGACTATAATATTCAGAAAGAGTCAACGCTGTACCTTGTATTGAGGCTCCGTGGGGCGATTATTGAACCTTCGTTGATGGCATTGGCTAGAAAGTATAACCAAGACAAGATGATTTGCAGAAAGTGCTATGCTCGGTTGCATCCTAGGGCAGTAAACTGCAGGAAAAAGAAGTGTGGCCATAGTAACCAGCCAAAGAAGAAGATCAAGTAAATCTGACATCTGAAGTGATCATATCGCTGGCTTATATTCACA

>*Cycas elongata Cyclophilin peptidyl-prolyl cis-trans isomerase CYP19-2*

AAAATCCCAGGGTGTTCTTGGACATCGCAATTGGTGGTGAGCCTGCAGGAAAAATTGTTTTGGAGCTTTTTGCAGATACTGCTCCTAAAACTACAGAAAATTTTAGAGCGCTTTGTAAAGGAGAGAAAGGTGTTGGAAAAACGACAAGGAAGCCTCTACATTACAAAGGGTCGATATTTCATCGTATTATTAAAGGCTTCATGGCCCAAGGAGGGGACTTTTCGAAGAGAGATGGCACTGGTGGAGAAAGCATCTATGGTGGTAAATTTGCAGATGAAAATGTCCGTCTCCGTCATGAGGGTCCTGGTGTTTTATCAATGGCAAATGCTGGTAGAAACACTAATGGTTCTCAGTTCTTCATTACCTTTGCATCTGCACCACATCTGGATGGGAAGCATGTTGTCTTTGGAAAGGTTGTAGAGGGAATGGATGTGTTGAAGAAAATTGAGCAGGTGCCCACTGATCATGGGAGGCCTACTTTTCCTGTTAAAATTGTAGACAGCGGTGAAAT
