# Supplementary material for: Selection and Validation of Reference Genes for qRT-PCR in Cycas elongata
Source: PLoS One. 2016 Apr 28;11(4):e0154384. doi: 10.1371/journal.pone.0154384 (PMC4849791; doi:10.1371/journal.pone.0154384)
Supplement: S1 Table — (DOC) [file pone.0154384.s006.doc]

S1 Table. 260/280 ratios and 260/230 ratios of all samples analyzed by ultraviolet spectrophotometer.

| **Samples** | **260/280 ratios** | **260/230 ratios** |
| --- | --- | --- |
| meg-A | 2.197 | 2.054 |
| mic-A | 2.009 | 2.121 |
| female-A | 2.102 | 2.129 |
| male-A | 2.195 | 2.202 |
| asexual-A | 2.148 | 2.189 |
| root-A | 2.457 | 1.959 |
| stalk-A | 2.401 | 1.903 |
| ovule-A | 2.060 | 2.137 |
| meg-B | 2.186 | 2.005 |
| mic-B | 1.954 | 2.159 |
| female-B | 2.119 | 2.144 |
| male-B | 2.140 | 2.161 |
| asexual-B | 2.027 | 2.200 |
| root-B | 2.349 | 2.066 |
| stalk-B | 2.498 | 2.196 |
| ovule-B | 2.039 | 2.173 |
