# Supplementary material for: Selection and Validation of Reference Genes for qRT-PCR in Cycas elongata
Source: PLoS One. 2016 Apr 28;11(4):e0154384. doi: 10.1371/journal.pone.0154384 (PMC4849791; doi:10.1371/journal.pone.0154384)
Supplement: S2 Table — (DOC) [file pone.0154384.s007.doc]

S2 Table. Raw Ct data used for statistical analysis in this study.

| Sample | ***CLATHRIN*** | ***PP2A*** | ***ACT7*** | ***RPB2*** | ***EF1*** | ***GAPC2*** | ***SAMDC*** | ***TIP41*** | ***MAPK*** | ***CYP*** | ***UBQ*** | ***EIF4*** | ***TUB*** |
| --- | --- | --- | --- | --- | --- | --- | --- | --- | --- | --- | --- | --- | --- |
| meg1 | 22.63 | 24.34 | 30.81 | 27.48 | 27.32 | 23.16 | 27.67 | 28.21 | 30.03 | 26.66 | 27.57 | 24.08 | 24.7 |
| meg2 | 22.33 | 24.65 | 30.47 | 27.28 | 27.16 | 24.05 | 27.54 | 27.85 | 30.07 | 26.48 | 27.39 | 23.95 | 24.4 |
| meg3 | 22.41 | 24.57 | 30.3 | 27.26 | 27.03 | 23.87 | 27.39 | 27.88 | 29.71 | 26.54 | 27.43 | 23.86 | 24.47 |
| meg4 | 24.38 | 23.62 | 29.68 | 26.67 | 24.85 | 23.19 | 27.01 | 26.96 | 28.69 | 26.36 | 26.51 | 23.24 | 24.09 |
| meg5 | 24.26 | 23.46 | 29.63 | 26.55 | 24.73 | 22.85 | 27 | 26.84 | 28.6 | 26.25 | 26.55 | 23.2 | 23.79 |
| meg6 | 24.3 | 23.51 | 29.81 | 26.5 | 24.79 | 23.08 | 26.92 | 26.94 | 28.69 | 26.27 | 26.65 | 23.26 | 23.99 |
| mic1 | 23.16 | 23.13 | 24.76 | 25.82 | 23.89 | 22.22 | 25.06 | 26.27 | 27.58 | 24.42 | 25.22 | 22.42 | 23.72 |
| mic2 | 22.95 | 22.65 | 24.49 | 25.5 | 23.61 | 22.11 | 24.62 | 26.11 | 27.41 | 24.29 | 25.03 | 22.25 | 23.38 |
| mic3 | 22.79 | 22.81 | 24.41 | 25.65 | 23.64 | 21.99 | 24.82 | 26.19 | 27.29 | 24.3 | 25.12 | 22.22 | 23.39 |
| mic4 | 23.68 | 23.47 | 27.76 | 26.3 | 24.36 | 22.86 | 25.81 | 26.46 | 27.96 | 25.51 | 25.97 | 22.64 | 24.22 |
| mic5 | 23.67 | 23.38 | 27.7 | 26.14 | 24.29 | 22.83 | 25.69 | 26.14 | 27.87 | 25.35 | 25.78 | 22.48 | 24.11 |
| mic6 | 23.73 | 23.15 | 28.04 | 26.32 | 24.29 | 22.93 | 25.87 | 26.33 | 27.96 | 25.5 | 26 | 22.62 | 24.11 |
| female1 | 21.7 | 21.88 | 22.38 | 24.77 | 22.82 | 20.88 | 25.72 | 24.3 | 27.15 | 23.06 | 22.63 | 22.4 | 22.99 |
| female2 | 21.39 | 21.61 | 22.1 | 24.41 | 22.55 | 20.67 | 25.45 | 24.08 | 26.78 | 22.84 | 22.3 | 22.17 | 22.66 |
| female3 | 21.46 | 21.52 | 22.11 | 24.46 | 22.42 | 20.69 | 25.51 | 24.03 | 26.76 | 22.78 | 22.32 | 22.15 | 22.73 |
| female4 | 21.65 | 21.69 | 22.19 | 24.61 | 22.02 | 21.35 | 25.47 | 23.95 | 26.82 | 22.9 | 22.59 | 22.05 | 23.24 |
| female5 | 21.53 | 21.4 | 22.06 | 24.49 | 21.76 | 21.24 | 25.35 | 23.94 | 26.72 | 22.77 | 22.52 | 21.98 | 23.07 |
| female6 | 21.92 | 21.62 | 22.26 | 24.81 | 22.07 | 21.45 | 25.63 | 24.01 | 26.89 | 22.97 | 22.65 | 22.26 | 23.3 |
| male1 | 22.14 | 21.34 | 24.37 | 25.62 | 20.81 | 22.25 | 25.63 | 25.15 | 27.67 | 23.54 | 23.68 | 22.13 | 21.99 |
| male2 | 21.77 | 21.01 | 23.98 | 25.32 | 20.51 | 22.05 | 25.37 | 24.92 | 27.31 | 23.26 | 23.31 | 21.71 | 21.64 |
| male3 | 21.82 | 21.11 | 23.86 | 25.27 | 20.46 | 21.81 | 25.4 | 24.93 | 27.26 | 23.3 | 23.32 | 21.68 | 21.59 |
| male4 | 21.5 | 21.62 | 22.53 | 24.36 | 21.68 | 21.12 | 24.04 | 24.23 | 26.83 | 23 | 23.59 | 21.68 | 23.03 |
| male5 | 21.38 | 21.43 | 22.4 | 24.28 | 21.6 | 21.22 | 24.08 | 24.25 | 26.83 | 23.04 | 23.55 | 21.82 | 23.01 |
| male6 | 21.45 | 21.62 | 22.44 | 24.5 | 21.65 | 21.2 | 24 | 24.25 | 26.88 | 22.96 | 23.72 | 21.74 | 22.97 |
| asexual1 | 23.13 | 23.1 | 23.57 | 25.89 | 24.03 | 22.09 | 25.16 | 25.41 | 28.48 | 23.12 | 23.27 | 23.3 | 23.08 |
| asexual2 | 22.92 | 22.89 | 23.28 | 25.81 | 23.97 | 22.09 | 25.01 | 25.25 | 28.18 | 23.07 | 23.23 | 23.22 | 22.87 |
| asexual3 | 22.46 | 22.53 | 22.83 | 25.28 | 23.42 | 21.64 | 24.54 | 24.72 | 27.62 | 22.58 | 23.04 | 22.64 | 22.41 |
| asexual4 | 22.14 | 21.63 | 23.32 | 25.46 | 21.31 | 22.59 | 26.06 | 25.32 | 29.06 | 24.4 | 26.43 | 24.19 | 22.92 |
| asexual5 | 22.09 | 21.52 | 23.3 | 25.35 | 21.26 | 22.49 | 26.1 | 25.37 | 29.29 | 24.46 | 26.21 | 24.06 | 22.93 |
| asexual6 | 22.31 | 21.85 | 23.59 | 25.75 | 21.52 | 22.72 | 26.24 | 25.53 | 29.38 | 24.59 | 26.18 | 24.28 | 23.08 |
| root1 | 22.73 | 22.15 | 27.04 | 25.32 | 23.29 | 21.93 | 24.87 | 25.33 | 26.96 | 24.5 | 25 | 21.62 | 23.11 |
| root2 | 20.7 | 20.88 | 21.38 | 23.77 | 21.82 | 19.88 | 24.72 | 23.3 | 26.15 | 22.06 | 21.63 | 21.4 | 21.99 |
| root3 | 20.39 | 20.61 | 21.1 | 23.41 | 21.55 | 19.67 | 24.45 | 23.08 | 25.78 | 21.84 | 21.3 | 21.17 | 21.66 |
| root4 | 20.46 | 20.52 | 21.11 | 23.46 | 21.42 | 19.69 | 24.51 | 23.03 | 25.76 | 21.78 | 21.32 | 21.15 | 21.73 |
| root5 | 20.65 | 20.69 | 21.19 | 23.61 | 21.02 | 20.35 | 24.47 | 22.95 | 25.82 | 21.9 | 21.59 | 21.05 | 22.24 |
| root6 | 20.53 | 20.4 | 21.06 | 23.49 | 20.76 | 20.24 | 24.35 | 22.94 | 25.72 | 21.77 | 21.52 | 20.98 | 22.07 |
| stalk1 | 20.92 | 20.62 | 21.26 | 23.81 | 21.07 | 20.45 | 24.63 | 23.01 | 25.89 | 21.97 | 21.65 | 21.26 | 22.3 |
| stalk2 | 21.14 | 20.34 | 23.37 | 24.62 | 19.81 | 21.25 | 24.63 | 24.15 | 26.67 | 22.54 | 22.68 | 21.13 | 20.99 |
| stalk3 | 20.77 | 20.01 | 22.98 | 24.32 | 19.51 | 21.05 | 24.37 | 23.92 | 26.31 | 22.26 | 22.31 | 20.71 | 20.64 |
| stalk4 | 20.82 | 20.11 | 22.86 | 24.27 | 19.46 | 20.81 | 24.4 | 23.93 | 26.26 | 22.3 | 22.32 | 20.68 | 20.59 |
| stalk5 | 20.5 | 20.62 | 21.53 | 23.36 | 20.68 | 20.12 | 23.04 | 23.23 | 25.83 | 22 | 22.59 | 20.68 | 22.03 |
| stalk6 | 20.38 | 20.43 | 21.4 | 23.28 | 20.6 | 20.22 | 23.08 | 23.25 | 25.83 | 22.04 | 22.55 | 20.82 | 22.01 |
| ovule1 | 22.52 | 23.59 | 26.25 | 26.17 | 27.5 | 22.64 | 27.06 | 25.77 | 30.23 | 25.66 | 26.16 | 22.94 | 24.17 |
| ovule2 | 22.55 | 23.21 | 25.71 | 25.88 | 26.93 | 22.44 | 26.84 | 25.3 | 29.76 | 25.59 | 25.88 | 22.62 | 23.84 |
| ovule3 | 22.62 | 22.88 | 25.16 | 25.37 | 26.36 | 22.17 | 26.42 | 25.09 | 29.09 | 25.32 | 26.26 | 22.28 | 23.61 |
| ovule4 | 22.08 | 22.75 | 26.27 | 25.89 | 24.57 | 22.25 | 26.18 | 25.64 | 28.29 | 25.13 | 24.43 | 22.37 | 22.69 |
| ovule5 | 21.88 | 22.57 | 26.32 | 25.92 | 24.58 | 22.14 | 26.24 | 25.6 | 28.34 | 25.15 | 24.31 | 22.4 | 22.58 |
| ovule6 | 22.14 | 22.72 | 26.45 | 26.11 | 24.75 | 22.43 | 26.52 | 25.86 | 28.59 | 25.31 | 24.6 | 22.71 | 22.81 |

Note: meg (megasporophyll ), mic (microsporophyll), female (female plant leaf), male (male plant leaf), asexual (asexual plant leaf ).

2 biological replicates and 3 technical replicates within each tissue type.
