# Supplementary material for: Selection and Validation of Reference Genes for qRT-PCR in Cycas elongata
Source: PLoS One. 2016 Apr 28;11(4):e0154384. doi: 10.1371/journal.pone.0154384 (PMC4849791; doi:10.1371/journal.pone.0154384)
Supplement: S3 Table — (DOC) [file pone.0154384.s008.doc]

S3 Table. Ranking of *Cycas elongata* reference genes calculated by geNorm, NormFinder and BestKeeper.

| **geNorm** | | **NormFinder** | | **BestKeeper** | |
| --- | --- | --- | --- | --- | --- |
| Gene | stability value | Gene | stability value | Gene | stability value |
| *GAPC2* | 0.32 | *PP2A* | 0.018 | *TUB* | 0.77 |
| *RPB2* | 0.32 | *RPB2* | 0.022 | *EIF4* | 0.78 |
| *TIP41* | 0.47 | *GAPC2* | 0.023 | *CLATHRIN1* | 0.86 |
| *PP2A* | 0.52 | *TIP41* | 0.024 | *SAMDC* | 0.90 |
| *EIF4* | 0.63 | *MAPK* | 0.038 | *GAPC2* | 0.91 |
| *MAPK* | 0.69 | *CYP* | 0.040 | *RPB2* | 0.92 |
| *CLATHRIN1* | 0.74 | *EIF4* | 0.048 | *PP2A* | 1.07 |
| *CYP* | 0.79 | *UBQ* | 0.049 | *MAPK* | 1.09 |
| *UBQ* | 0.85 | *CLATHRIN1* | 0.052 | *TIP41* | 1.13 |
| *TUB* | 0.92 | *TUB* | 0.071 | *CYP* | 1.37 |
| *SAMDC* | 0.98 | *SAMDC* | 0.071 | *UBQ* | 1.67 |
| *ACT7* | 1.16 | *EF1* | 0.121 | *EF1* | 1.85 |
| *EF1* | 1.29 | *ACT7* | 0.151 | *ACT7* | 2.37 |
